# Home range overlap between small Indian mongooses and free roaming domestic dogs in Puerto Rico: implications for rabies management

**DOI:** 10.1038/s41598-023-50261-7

**Published:** 2023-12-22

**Authors:** Caroline C. Sauvé, Are R. Berentsen, Steven F. Llanos, Amy T. Gilbert, Patrick A. Leighton

**Affiliations:** 1 Faculté de Médecine Vétérinaire, Université de Montréal, 3200 Rue Sicotte, Saint-Hyacinthe, QC J2S 2M2 Canada; 2 United States Department of Agriculture, Animal and Plant Health Inspection Service, Wildlife Services, National Wildlife Research Center, 4101 LaPorte Avenue, Fort Collins, CO 80521 USA; 3 United States Department of Agriculture, Animal and Plant Health Inspection Service, Wildlife Services, National Wildlife Research Center, PO Box 38, Lajas, PR 00667 USA

**Keywords:** Behavioural ecology, Ecological epidemiology, Invasive species, Tropical ecology, Infectious diseases, Animal behaviour

## Abstract

The small Indian mongoose (*Urva auropunctata*) is the primary terrestrial wildlife rabies reservoir on at least four Caribbean islands, including Puerto Rico. In Puerto Rico, mongooses represent a risk to public health, based on direct human exposure and indirectly through the transmission of rabies virus to domestic animals. To date, the fundamental ecological relationships of space use among mongooses and between mongooses and domestic animals remain poorly understood. This study is the first to report mongoose home range estimates based on GPS telemetry, as well as concurrent space use among mongooses and free roaming domestic dogs (FRDD; *Canis lupus familiaris*). Mean (± SE) home range estimates from 19 mongooses in this study (145 ± 21 ha and 60 ± 14 ha for males and females, respectively) were greater than those reported in prior radiotelemetry studies in Puerto Rico. At the scale of their home range, mongooses preferentially used dry forest and shrubland areas, but tended to avoid brackish water vegetation, salt marshes, barren lands and developed areas. Home ranges from five FRDDs were highly variable in size (range 13–285 ha) and may be influenced by availability of reliable anthropogenic resources. Mongooses displayed high home range overlap (general overlap index, GOI = 82%). Home range overlap among mongooses and FRDDs was intermediate (GOI = 50%) and greater than home range overlap by FRDDs (GOI = 10%). Our results provide evidence that space use by both species presents opportunities for interspecific interaction and contact and suggests that human provisioning of dogs may play a role in limiting interactions between stray dogs and mongooses.

## Introduction

The small Indian mongoose (*Urva auropunctata*) is a small opportunistic omnivore introduced to sugarcane-producing islands worldwide during the late nineteenth and early twentieth centuries to limit agricultural crop damage by invasive rodents^1,2^. Within 10 years of its introduction to Jamaica, a precipitous decline in native fauna was noticed because of predation by mongooses, which are now considered a pest species throughout much of their introduced range^3^. Mongooses are the primary wildlife reservoir for canine rabies on at least four Caribbean islands: Puerto Rico, Grenada, Hispaniola, and Cuba^4^. They may also be important in the transmission of other zoonoses like leptospirosis^5–8^, bartonellosis^9^, salmonellosis and *Campylobacter* spp^10^.

The first laboratory confirmed case of rabies in mongooses in the Western hemisphere was in Puerto Rico in 1950^11^. Following the initial mongoose case, several cases of rabies in domestic animals were reported across the island over a period of 3 months. All suspect animals were reported to have had contact with mongooses prior to displaying clinical signs of rabies infection^11^. Similarly, in Grenada and Cuba, rabies cases associated with mongoose interaction were laboratory confirmed in 1956, while it took until the 1980s to recognize the importance of rabies in mongooses in the Dominican Republic^12^. Across the islands where mongoose rabies is enzootic, phylogenetic reconstruction indicates a complex history of independent introductions of cosmopolitan canine rabies virus which happened after the introduction and establishment of mongoose populations across islands^13^. Rabies has remained enzootic on these islands despite intensive dog (*Canis lupus familiaris*) vaccination and prior rabies control focused on local mongoose population reduction has been largely unsuccessful^12,14^.

During 2021, mongooses accounted for over 70% of reported animal rabies cases in Puerto Rico, whereas domestic dogs represented 18% of cases^15^. Human fatalities from rabies virus infection, transmitted directly through mongoose bites or bites from rabid dogs which were observed to have been bitten by a mongoose, have been reported in the twenty-first century (e.g.^15,16^), confirming the continued threat to public health and highlighting the potential role of domestic dogs as vectors for the transmission of mongoose rabies virus to humans. The dynamics of rabies virus transmission among mongooses and from mongooses to domestic animals remain poorly understood (but see^17^). The spillover rate from the mongoose reservoir to domestic animals is considered low^18^. However, in Puerto Rico, large feral dog populations, in addition to unrestricted domesticated or stray animal movements and the absence of compulsory vaccination for domestic animals, all serve to increase opportunities for contact between mongoose and susceptible domestic animal populations.

To gain insight into intra- and interspecific potential for rabies virus transmission among mongooses and between mongooses and dogs, it is necessary to understand the fundamentals of resource and space use for both species. Home range (HR) estimators are widely used to measure animal space-use patterns and represent the spatial extent of an individual’s location and movements during the course of daily foraging and/or refuging activities^19^. Animal space use and movement patterns are fundamental components in habitat and resource selection studies, population dynamics, gene flow, and intra- and interspecific interactions. Spatial and temporal habitat use overlap and habitat selection can provide valuable information on when and where infectious disease transmission between species or populations is most likely to occur, as well as assist managers in developing disease mitigation strategies (e.g.^20,21^).

Few studies on mongoose HR have been conducted in Puerto Rico, employing a variety of field methods and estimators^5,22,23^, and no ecological data is available for free-roaming domestic dogs (FRDD) on the island. Traditional capture-mark-recapture (CMR) and VHF radio telemetry are relatively inexpensive with respect to materials, yet they tend to be time and labor intensive and may not provide fine scale movement data required for accurate HR estimates and resource selection studies. In addition, both CMR and VHF telemetry focus detection on specific spatial areas of interest and can underestimate long-range movements when individuals are not successfully recaptured or relocated. The use of Global Positioning System (GPS) for tracking wildlife movements has grown over the past several decades, and a comparative study where both GPS and VHF telemetry were deployed concurrently on Florida panthers revealed that all GPS-derived HR estimates performed better based on area under the curve^24^ than VHF-derived estimates^25^. Despite broad interest in the fine-scale, detailed movement data for wildlife available through GPS telemetry, equipping small (e.g., 0.5 kg or less) animals like mongooses with GPS tags was challenging during the last decade due mostly to the device size and attachment methods. However, as GPS devices became smaller, collars carrying this technology suitable for attachment to mongooses became available, opening new possibilities for mongoose space-use, movement, and interspecific contact studies.

Our objectives were to use GPS collars on mongooses and FRDDs to (1) estimate mongoose HR sizes, (2) contrast the spatial overlap between mongooses and FRDDs, and (3) identify fine-scale mongoose habitat preferences to gain a better understanding of potential inter- and intraspecific interactions between mongooses and FRDDs, with implications for rabies virus transmission and potential disease mitigation measures.

## Results

### Home range estimation

**Figure 1:**
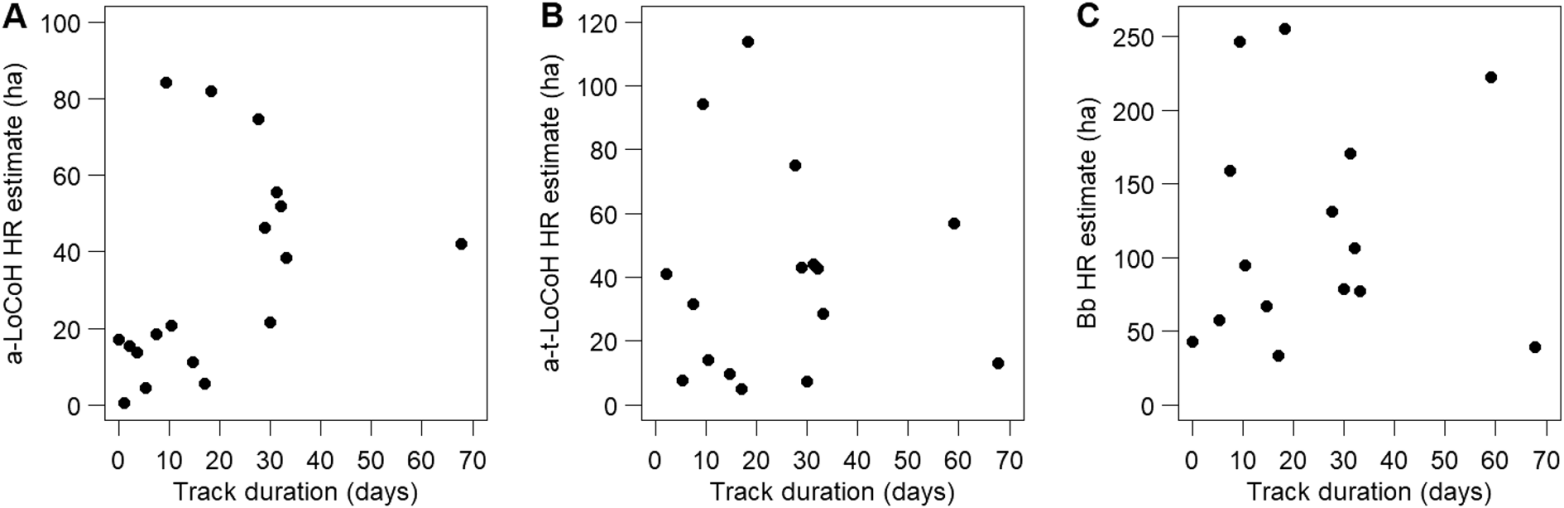
The relationship between small Indian mongoose (*Urva auropunctata*, n = 19) home range (HR) size and GPS track duration (days), by HR estimator [(**A**) local convex hulls, (**B**) time-dependent local convex hulls, and (**C**) Brownian bridge 95% isopleth].

We collared 23 mongooses (four females and 19 males). Using the abundance estimate derived from a previous CMR in the fall of 2021 (N = 49; 95% CI = 43–56 mongooses; USDA, Unpublished data), the tagged proportion of animals may represent ~ 47% of the estimated population over the study site. However, given seasonal variability reported in mongoose population densities (e.g.^26,27^), the estimated population abundance should be interpreted with caution. Of the 23 collared mongooses, one mongoose was recaptured three days later and killed in the trap by a FRDD and one mongoose slipped its collar four days post-deployment. In both cases, we detected the mortality signal using the VHF receiver, recovered the collar, and re-deployed these collars on new mongooses. We remotely downloaded the tracking data from 19 collared mongooses and obtained tracking data of mongoose movements, with a duration from 3 h and up to 68 days per individual (mean 21.02 ± 4.33 days; Table 1). To test for biases in HR size associated with the number of locations obtained per mongoose, we generated an area-track duration curve. There was no effect of individual track duration on HR size among individuals (Fig. 1). Home range sizes estimated using a-LoCoH, t-LoCoH and the Bb were correlated (r > 0.70; Supplementary Fig. 1). While a-LoCoH and t-LoCoH estimates did not differ significantly (slope 95% CI = 0.75–1.42; intercept 95% CI = − 18.96 to 12.39), Bb estimates were, on average, two- and threefold larger than a-LoCoH and t-LoCoH estimates, respectively. On average, mongoose male HRs were larger than female HRs, with the difference between sexes varying between a 2.4 and a 6.3 multiplying factor depending on the HR estimator used (Table 2).

**Table 1:** Summary of track duration, and home range (HR) and core area (CA) sizes from 19 small Indian mongooses (M01 to M19; *Urva auropunctata*) and five free-roaming domestic dogs (D01 to D05; *Canis lupus familiaris*) equipped with GPS collars in southern Puerto Rico. equipped with GPS collars in southern Puerto Rico. HR and CA were derived from three different estimators, namely time-independent and time-dependent local convex hull (a-LoCoH and t-LoCoH) 95% isopleths, and Brownian bridge (Bb) 95% and 50% kernels.

Animal ID	Sex	No. Fixes in GPS track	Track duration (d)	a-LoCoH HR area (ha)	t-LoCoH HR area (ha)	Bb 95% UD HR area (ha)	Bb 50% UD CA area (ha)
M01	M	105	10.35	20.8	14.1	94.5	16.6
M02	M	851	59.02	264.1	56.8	222.5	16.5
M03	M	83	9.44	84.2	94.4	246.7	57.6
M04	F	298	30.04	21.5	7.4	78.9	7.9
M05	M	278	31.17	55.4	44.0	170.5	24.3
M06	M	1158	67.75	42.2	13.0	39.7	6.0
M07	M	209	29.00	46.3	43.2	288.6	33.0
M08	M	564	33.10	38.3	28.6	77.2	6.7
M09	M	196	18.29	81.8	113.8	255.2	50.9
M10	M	537	27.58	74.7	75.1	131.0	30.7
M11	M	6	3.00 h	17.0	–	42.7	10.5
M12	M	348	32.15	51.87	42.8	106.7	20.9
M13	M	60	5.31	4.3	7.6	57.3	7.1
M14	M	41	2.17	15.4	40.9	–	18.6
M15	F	188	17.04	5.6	5.1	33.3	5.1
M16	M	89	7.54	18.5	31.6	159.2	25.7
M17	F	197	14.62	11.2	9.8	66.9	8.7
M18	M	13	3.65	13.7	–	–	23.2
M19	M	9	1.06	0.6	–	–	10.5
D01	F	1877	64.21	77.2	55.4	80.0	15.2
D02	M	2140	61.67	126.0	8.5	40.2	2.5
D03	F	2246	67.60	12.7	3.8	11.0	1.3
D04	F	2318	71.17	263.2	190.6	187.2	10.8
D05	M	1660	53.73	284.6	209.2	220.6	27.4

**Table 2:** Overall and sex-specific average (± SE; 95% confidence interval in parentheses) small Indian mongoose (*Urva auropunctata*, n = 17) and free-roaming domestic dog (FRDD, *Canis lupus familiaris*; n = 5) home range size. Home ranges were derived from three different estimators, namely time-independent and time-dependent local convex hull (a-LoCoH and t-LoCoH) 95% isopleths, and Brownian bridge (Bb) 95% kernel. All home range sizes presented are in hectares. A two-sided t-test was performed to assess for differences in home range size between the sexes, and the *P*-value from this test is presented.

Method	Both sexes	Males	Females	*P*-value for difference between sexes
Mongoose				
a-LoCoH	46 ± 14 (17–74)	51 ± 16 (18–85)	13 ± 5 (0–33)	0.029
t-LoCoH	39 ± 8 (22–57)	47 ± 8 (27–66)	7 ± 1 (1–13)	< 0.001
Bb 95%	129 ± 21 (84–174)	145 ± 21 (94–197)	60 ± 14 (1–118)	0.008
Dogs				
a-LoCoH	153 ± 53 (7–299)	205 ± 79 (0–1213)	117 ± 75 (0–441)	0.490
t-LoCoH	93 ± 44 (0–217)	109 ± 100 (0–1383)	83 ± 56 (0–323)	0.848
Bb 95%	108 ± 41 (0–222)	130 ± 90 (0–1276)	93 ± 51 (0–313)	0.757

**Figure 2:**
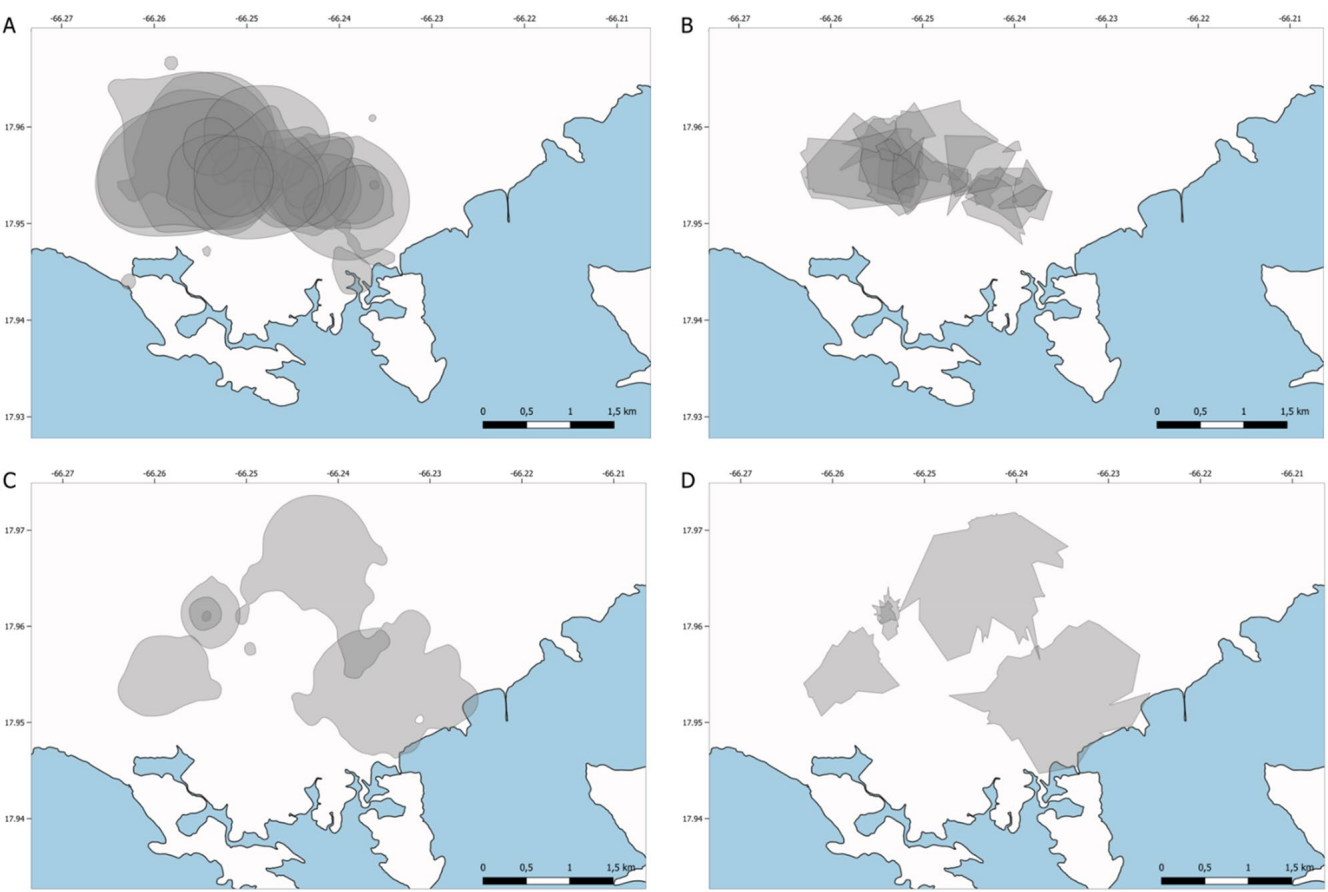
Small Indian mongoose (*Urva auropunctata,* panels (**A**) and (**B**)] and free-roaming domestic dog [FRDD, *Canis lupus familiaris*; panels (**C**) and (**D**)] home ranges derived from Brownian bridge (**A**,**C**) and time-dependent local convex hulls (**B**,**D**) illustrating the intraspecific home range overlap among collared mongooses (n = 19) and separately among FRDDs (n = 5).

Estimates of mongoose intra-specific HR overlap also varied based on the estimator used (Fig. 2). The mongoose intra-specific GOI calculated using Bb was 82.4%, while the GOI measured using the t-LoCoH HRs was 70.7%. Pairwise percent overlaps among individual mongooses ranged between 0 and 100% (median: 18.8%) and 0 and 87% (median: 0%) for Bb and t-LoCoH HR estimates, respectively (Supplementary Table 1).

We collared six FRDDs (four females and two males) and recovered data from five, whereas one FRDD was never relocated following initial capture and collaring. We obtained tracking data of durations that largely exceed the mongoose tracking durations and so we subset the FRDD tracking data that temporally coincided with mongoose tracking data. This resulted in FRDD track durations ranging between 53.73 and 71.17 days, with no correlation between track duration and HR size estimate (Table 2). FRDD HR sizes derived from the three estimators were highly correlated (r > 0.90; Supplementary Fig. 1) and did not differ. Within any given estimator, there was no difference in FRDD HR size between sexes (Table 2).

**Figure 3:**
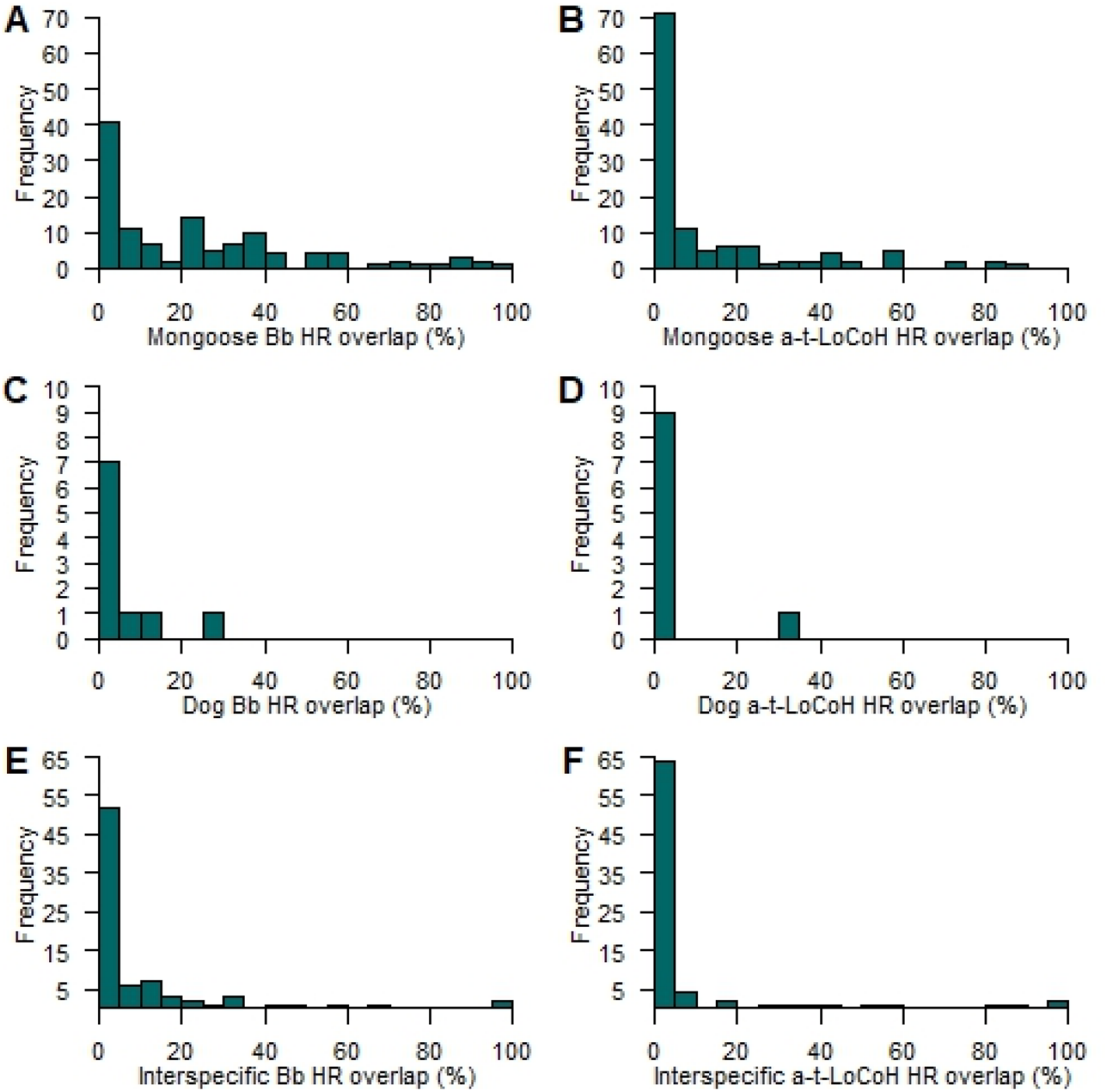
Distributions of pairwise percent home range (HR) overlap among small Indian mongooses [*Urva auropunctata*; (**A**,**B**)], free-roaming domestic dogs [FRDD, *Canis lupus familiaris*; (**C**,**D**)], and between mongooses and FRDDs (**E**,**F**) calculated using a Brownian bridge 95% isopleth (Bb; **A**,**C**,**E**) and time-dependent local convex hulls (t-LoCoH; **B**,**D**,**F**).

The two FRDDs (one male and one female) with the lowest HR sizes were collared by hand-feeding them from a dirt road, near residences where they were routinely observed. We captured and collared the four other FRDDs in cage traps and had not previously observed them in proximity to residences within our study site. Differences in HR size estimates between docile hand-fed FRDDs versus those cage-trapped are only marginally different (p = 0.095 and 0.072 for t-LoCoH and Bb estimates, respectively), which suggests that reliance on human provisioning may have a greater influence than animal sex on FRDD HR. The FRDD intra-specific GOIs were 10.4% and 1.2% for Bb and t-LoCoH HR estimates, respectively. The Bb and t-LoCoH generated HRs with pairwise percent overlap among FRDDs ranging between 0 and 27% (median: 0%) and 0 and 33% (median: 0.8%), respectively (Fig. 3; Supplementary Table 1). FRDD median intraspecific Bb HR percent overlap was smaller than mongoose intraspecific HR overlap (Bb: *P* = 0.008), while t-LoCoH HR percent overlap did not differ among species (*P* = 0.447).

Interspecific pairwise HR overlap between FRDDs and mongooses ranged between 0 and 100% for both Bb and t-LoCoH estimates, while the median interspecific overlap was greater for Bb HRs than for t-LoCoH HRs (2.2% and 0%, respectively; *P* < 0.001). Median interspecific pairwise HR overlap was smaller than mongoose intraspecific HR overlap when estimated using the Bb (*P* < 0.001), but identical with the t-LoCoH methods. In contrast, interspecific was greater than FRDD intraspecific pairwise HR overlap using the Bb (*P* = 0.007) but equivalent using the t-LoCoH (*P* = 0.087) HRs. Interspecific GOI were 50.00 and 51.73% for Bb and t-LoCoH, respectively.

### Resource selection function (RSF)

From 100 correlated random walk tracks for each of the 19 tracking records recovered from collared mongooses, we discarded ‘available’ locations situated in open water (n = 85,295; 16.2%) because these do not represent available habitat for our terrestrial species of interest. In addition, we removed available locations from extremely rare land cover types (< 0.005%) which were not represented in ‘use’ locations and caused convergence issues in the RSF (total removals: 144 from six landcover types). This resulted in 5224 pairs of use (n = 1 location/pair) and available (range = 57–100; average = 83.6 ± 0.2 locations/pair) points associated with either one of 20 land-cover categories.

**Figure 4:**
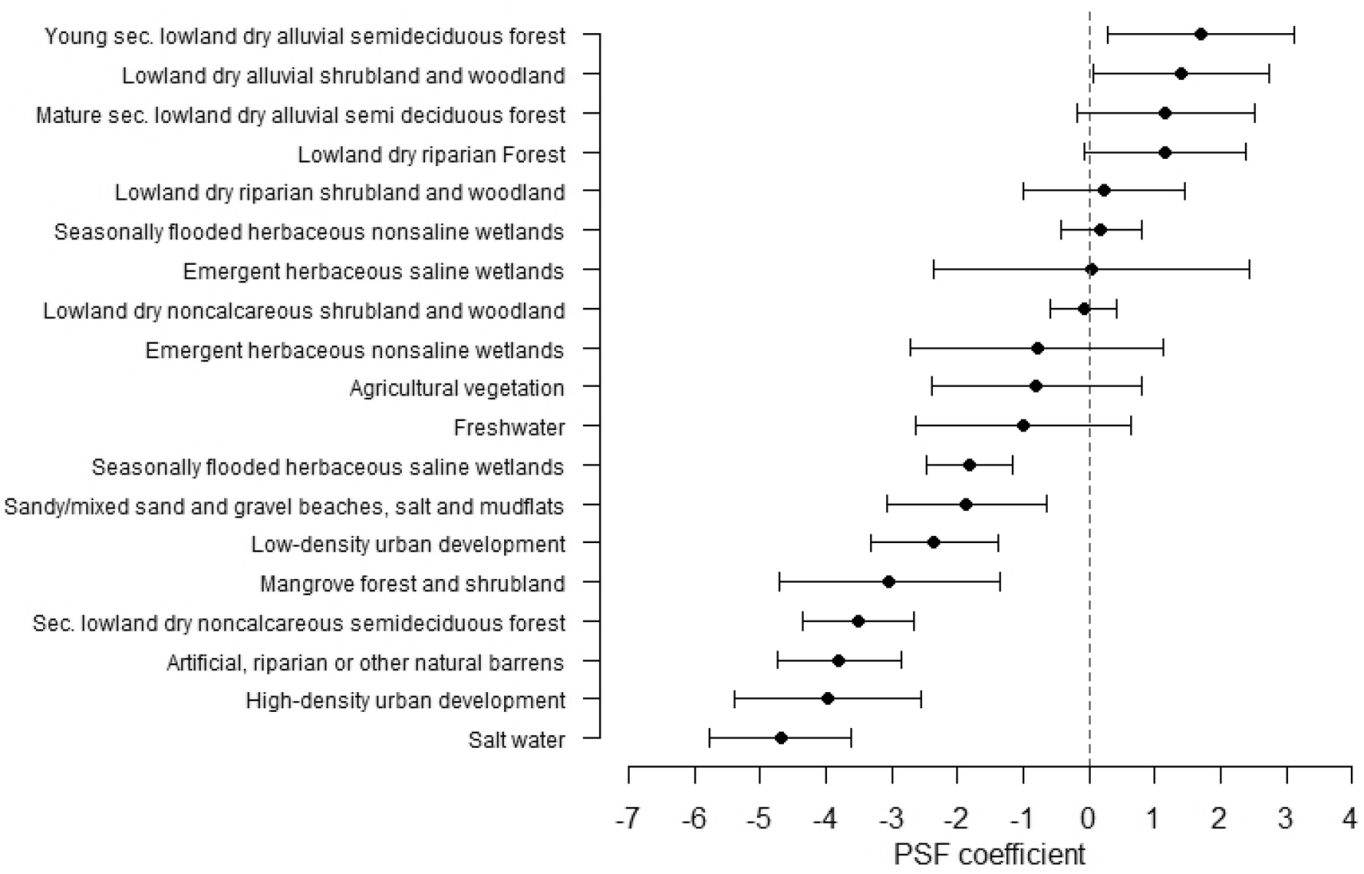
Coefficients (black circle) and their 95% confidence interval (black whiskers) from a path selection function (PSF) fitted using a condition logistic regression with animal ID as a cluster and the most represented land cover class (grasslands and pastures) set as the reference level. Land cover classes having a positive and negative coefficient were preferred and avoided by small Indian mongooses (*Urva auropunctata*), respectively, compared to grass and pastures.

We fitted a conditional logistic model to our paired use/available data and categorical covariates and obtained adequate model goodness of fit (concordance = 0.78 ± 0.07; Wald test χ^2^ = 27,476,480; df = 19, *P* < 0.001). Mongooses preferred lowland dry alluvial semideciduous forest, shrubland and woodland over the most common land-cover type over the study area (i.e., dry grasslands and pastures; Fig. 4). In contrast, mongooses avoided mangroves, seasonally flooded saline wetlands, beaches, urban developments, and barrens (Fig. 4). Other land-cover types, including non-saline wetlands, lowland dry riparian or noncalcareous shrubland and woodland, agricultural vegetation, and freshwater were not selected nor avoided compared to dry grasslands and pastures (Fig. 4).

## Discussion

This study is the first to report mongoose home range estimates based on GPS telemetry, as well as concurrent space use among mongooses and FRDDs. Mongoose space use is of interest due to their roles as a pest species in tropical islands across the globe, and as a wildlife reservoir for canine rabies in four Caribbean islands. We calculated mongoose and FRDD HR using three estimators that capture the temporal dependence in animal tracking data, which will favor comparison with future small Indian mongoose HR studies across their introduced and native ranges. Mongooses displayed high intraspecific HR overlap, and intermediate HR overlap with FRDDs, providing evidence that space use by both species presents opportunities for interspecific interaction. Domestic dogs can act as an intermediate between wildlife and humans for the transmission of infectious diseases, including rabies. The differential overlap in space use between mongooses and stray compared to feral dogs suggests that dog behaviour and human provisioning of dogs may play a role in limiting interactions between stray dogs and mongooses.

The HRs derived from both local convex hull estimators (a-LoCoH and t-LoCoH) were highly correlated, since they both rely on convex hulls created using nearest neighbors, although defined by different criteria^28^. In contrast, the Bb estimator assumes a conditioned random walk between successive locations and estimates the probability that the individual occurred in an area during a given time interval^29^. Interestingly, our t-LoCoH and Bb HR size estimates, which both explicitly account for time between successive fixes, were more strongly correlated than our a-LoCoH (implicit capture of time dependence). This suggests that our 30 min GPS sampling interval introduced substantial time dependence in the location data and justifies the relevance of using time-explicit HR estimators. Similarly, estimators incorporating a temporal dependence component performed better for estimating Florida panther home rage (GPS collar sampling interval range: every 1 to 7 h) regardless of the underlying estimator types^25^.

Bb movement models account for spatial uncertainty of every location and potential movement between successive locations^30^. Thus, they generally produce HRs that are more connected and have smoother boundaries than convex hull approaches. The correlation among the Bb and t-LoCoH HR estimates were greater for FRDDs than mongooses. Moreover, while there was a threefold difference between t-LoCoH and Bb mongoose HR size estimates, there were no significant differences between size estimates for FRDD HRs. This likely results from the longer and more regular tracking records obtained for FRDD compared to mongooses, since Bb estimates are more robust to irregularly sampled data^30^. The additional locations in FRDD track records likely ‘filled-in’ the gaps in intermediate locations that may be missing for some estimators to adequately capture space use by mongooses. Hereafter, we discuss findings related to mongoose and FRDD HRs considering only the Bb estimates.

Previous small Indian mongoose studies derived HR size from mark-recapture trapping data, homing, and/or VHF telemetry (see Table 1 in ^23^). There is substantial variability among estimates reported in these studies (average mongoose HR estimates range: 0.75–191 ha; see^23^), which are partly attributable to differences in tracking methods and HR estimators used. This study is the first to estimate mongoose HR using GPS telemetry and estimators incorporating temporal dependence assumptions in the data. The mongoose HR sizes reported in the present study (mean ± SE: 145 ± 21 ha and 60 ± 14 ha for males and females, respectively) are considerably larger than those reported in a recent VHF study conducted in Puerto Rico (means: 43–56 ha for males and 27–30 ha for females, depending on estimator considered^23^). In conventional VHF telemetry studies, animals must be re-located by personal scanning the study area. Unsuccessful relocation of every collared mongoose during each tracking attempt are likely due to animals temporarily leaving the study area, which may result in underestimation of HR sizes using that method^23^. In addition, VHF tracking generally results in comparatively fewer locations assumed to represent the space use of the animal, and a greater number of locations can translate into larger HR estimates^31^. In Puerto Rico, where mongoose HR estimates may be used to guide disease or damage mitigation strategies, underestimating HR sizes may result in inefficient mongoose rabies control strategies. The gains in data quality provided by GPS technology may be worth the cost of this comparably more expensive method when estimating mongoose HR in relation to damage and/or disease management.

A recent mongoose VHF study on Puerto Rico demonstrated that mongoose HR vary by season, sex and site sampled regardless of the estimator used^23^. As typically seen in mammals, male HRs from the present study were, on average, larger than female HRs and consistent with sexual dimorphism in this species. Sex-biased differences had varying degrees of support across most mongoose HR studies conducted in the Caribbean and other tropical islands (see^23^), whereas there was no sex-specific difference in HR size in mongooses in their native range of India^32^. In non-territorial species, resource availability, including food, is considered the ultimate determinant of both population density and HR size^33^. For several carnivore species, population density increases while HR size decreases along the natural-urban habitat gradient^34^. Berentsen et al.^23^ suggested that the differences in HR sizes observed among the sites they sampled might be due to the relative proximity to anthropogenic resources. We conducted the present study over a single site and season and cannot assess the effects of habitat, density, and season on the results. Recent studies revealed important habitat-specific differences in mongoose densities in their non-native range in the Caribbean [26, USDA, Unpublished data]. Conducting comparable (i.e., in terms of study design and analytical estimators used) studies estimating both HR size and population density in multiple habitat types and seasons would provide unprecedented information regarding the relationship between resource availability, mongoose demography, and space use.

Published studies on FRDD HRs are scarce and are mainly from regions where canine rabies is endemic^35,36^. The mean HR reported for 135 FRDDs in Indigenous communities in tropical northern Australia was estimated at 7 ha^36^. However, some FRDDs roamed considerably more, and exhibited HR sizes of 40–104 ha^37^. Meek^38^ also reported considerable differences in mean HR sizes for FRDDs that undergo wandering forays (927 ha) and sedentary FRDDs (2.6 ha) in southern Australia. In northern Australia, FRDD HRs were reported to vary between communities and regions, to be larger before compared to following the wet season, and larger for males than females^36^. More generally, HR sizes tend to be smaller for FRDDs living close to or within residential settings in urban and suburban areas across several cities of the United States and in Naples, Italy^39^. We found greater than tenfold variation in FRDD HR sizes (range 11–221 ha). Our sample size is small, limiting inference regarding the effects of most covariates on HR size. Nevertheless, we observed a marked dichotomy in FRDD behaviour on the site; some tolerated human presence and begged for food (hereafter referred to as stray FRDD), while others could only be approached following capture in cage traps, were skittish and avoided human presence (hereafter referred to as feral FRDD). The two collared stray FRDDs had the smallest HR sizes (11 and 40 ha) and were repeatedly observed resting in the shade of human residences. Moreover, the FRDD with the smaller HR was a female that nursed a litter of young in an unfenced residential yard. In contrast, feral FRDDs had larger HRs (range 80–221 ha). While they may have been opportunistically observed within the study area, the feral FRDDs did not shelter in the vicinity of human residences. As described elsewhere, we found suggestive evidence that fidelity to anthropogenic resources may represent a major driver of FRDD space use in rural Puerto Rico, with potential impacts for rabies virus transmission between mongooses and dogs.

In this study, we restricted the period of FRDD tracking records analysed to match that of mongoose collars, as the mongoose collars had a much shorter battery life. However, dog collars recorded data for > 7 months. While the HR for most FRDDs remained essentially unchanged after the period covered by the present study, one male FRDD dispersed ~ 10 km from the centroid of the HR (Supplementary Fig. 2). Upon initial capture, that FRDD was guarding a litter of pups within the study area. When he was resighted for data download 7 months later, he was guarding a different litter within his new area. Similarly, male FRDD have been reported to remain in contact with their litter for the first 6–8 weeks and may provide parental care to pups by regurgitation^40^. FRDD population social structure and reproductive behaviour thus influence space use and HRs, which are dynamic over the life of individuals.

Home range overlap indices can be used as proxies for assessing the degree of interaction among individuals. Most overlap indices available in the literature rely on the use of pairwise comparisons among individuals^41^. However, when comparing a high number of individuals, interpretation of overlap matrices becomes challenging, as measures of central tendency (such as means or medians) can be unrepresentative of the distribution of pairwise overlap indices^42^. In this study, we computed pairwise intra- and interspecific percent HR overlap among tagged individuals, as well as the newly proposed general overlap index (GOI^42^), which represents a simple and straightforward measure of HR overlap. However, while pairwise percent overlap are robust to sample size if tracked individuals are sampled randomly over the study area, GOI can be affected by sample size if tracking a higher percentage of the population results in a higher number of overlapping home ranges over a limited total area.

There was considerable variation in intraspecific pairwise percent overlap among collared mongooses (range: 0–100%, median: 19%). This finding is similar to^32^, who reported HR overlap ranging between 2–96% (average: 51%) among nine small Indian mongooses radio-tracked in their native range over ~ 430 ha in the village of Chandrabani, India. Although not quantified, mongoose HR overlap was also reported in a previous study conducted in southern Puerto Rico^23^. In northeastern Puerto Rico^22^ suggested a single mongoose may share range with up to 29 other mongooses. In the present study and a study in their native range^32^, individual mongoose HR overlapped substantially both within and between sexes. The high mongoose intra-specific GOI (82%) provides overall, quantitative evidence that mongoose HRs demonstrated considerable overlap, which supports the notion that mongooses are non-territorial^43,44^. A high level of space use overlap among individuals allows mongooses to reach very high population densities in certain habitats (e.g.^14,26^). Habitat-specific population densities and the degree of interaction among individuals were suggested as important drivers of mongoose rabies dynamics in Puerto Rico^17^. Conducting HR studies using standardized methods across a range of habitat types and mongoose densities would provide valuable information regarding the relationships between mongoose demography, space use, and inter-individual interactions to inform the design of rabies control programs.

Very few studies report HR overlap among FRDDs. One study on a university campus in Brazil qualitatively reported that HR from docile FRDDs overlapped in areas of high human traffic, whereas the HR of individuals from an elusive pack did not overlap with that of other FRDDs frequenting the campus^45^. FRDDs form social structures made of an aggregation of breeding pairs and their kin (offspring and subadults), where members of the different breeding pairs are generally not related^39^. The degree of interactions among individuals and of HR overlap in FRDDs are thus expected to vary within and between social groups and their reliance on human resources. In the present study, we intended to collar FRDDs from different social groups, which is supported by the low measured (range 0–33%, median 0%) pairwise intraspecific percent HR overlap and GOI (10%). Although sample size in this study is limited, which could bias the GOI, the fact that both pairwise percent HR overlap and GOI are smaller among FRDDs than mongooses suggest that social groups of FRDDs may have a lower degree of intraspecific interactions than individual mongooses. Increasing sample size, tracking animals within and across social groups, and conducting similar studies across the rural–urban gradient would provide a better representation of space use and intraspecific interaction dynamics, as well as of the influence of anthropogenic resources among FRDDs in Puerto Rico. This information could be used to identify differential potential for contribution in infectious disease spread among FRDDs (e.g.^46,47^), and help target rabies control interventions.

This study is the first to concurrently assess mongoose and FRDD HRs, and therefore quantify HR overlap between the two species. There was a wide range (0–100%) of pairwise percent overlap between mongoose-FRDD pairs. Interestingly, the percent HR overlap measured among mongooses and stray FRDDs was all < 20%, while HR overlap up to 100% was observed among mongooses and feral FRDDs. Closer proximity to human residences may provide FRDDs some protection against interactions with mongooses and potential exposure to this wildlife rabies reservoir. This protective effect could further expand to human-FRDD interactions and risks for rabies transmission, if feral FRDDs that have the highest risks of exposure to mongoose rabies generally tend to avoid human presence. Nevertheless, rabies-induced behavioural alterations may thus override apparent ecological segregation between stray FRDDs and mongooses.

The degree to which the overlap in space use reported in this study can predict the frequency of interspecific interactions which are significant for infectious disease spread requires further investigation and depends on the mode of transmission of the infectious agents. In the case of rabies virus, since it is transmitted through direct contact with saliva or nervous system tissue, direct interactions (e.g., agnostic behavior, predation) are necessary for the virus to spillover from the mongoose reservoir to FRDDs. Studies investigating the fine scale spatiotemporal overlap between mongoose and FRDDs are thus required to assess epidemiologically significant interspecific encounter rates in the context of rabies virus transmission. Nevertheless, the results from this study demonstrate a non-negligible overlap in mongoose and FRDD spatial use, and warrants caution related to a potential risk for mongoose rabies to spillover into FRDDs. Responsible companion dog ownership, including restricting the movement of dogs and maintaining up to date rabies vaccination, remain the best measures to mitigate human risks of contracting rabies through exposure to domesticated dogs^48^.

There is important fine-scale variation in land cover composition over the site, which are captured by the 15-m resolution PRGAP Landcover data^49^ used in the resource selection analysis conducted in this study. When trapping mongooses, some trap locations often yield large number of captures, while others are less frequented^26^ [A.R.B. and C.C.S., pers. obs.]. This suggests differential spatial use within the habitat by mongooses at a scale that cannot be captured by the typically used 100 m × 100 m trapping grid or by VHF telemetry. The reduced location error and relatively high sampling rate of the GPS tracking data collected during this study allowed an unprecedented fine-scale investigation of habitat selection in small Indian mongooses. Compared to the land cover class most readily available over the study area (dry grasslands and pastures), mongooses selected dry forests and shrublands, while they avoided saltwater-flooded areas and urban developments. Interestingly, measured mongoose preferences and avoidances corresponded particularly closely to the relative population densities reported on St. Kitts, where mongooses were ~ 2.5 times more abundant on a dry forest/shrubland site and ~ 3.6 times less abundant on a suburban site compared to a grassland site^26^. This suggests that habitats supporting high mongoose densities might contain resources that are selected at finer spatial scales by individuals. Conducting similar GPS studies in other habitats would allow exploring this potential relationship between mongoose local density and fine-scale resource selection within their HR. Such studies may be useful to develop predictive tools to identify high-use areas where mongooses converge, which may also potentially represent hotspots for intraspecific infectious disease transmission. Likewise, preferred land cover types may be targeted to optimize wildlife management interventions, such as oral rabies vaccination or baiting for population reduction.

## Conclusion

This study reports the first deployment of GPS tracking units on small Indian mongooses, as well as the first spatiotemporally concurrent tracking of mongooses and FRDDs. Spatial heterogeneity in habitat carrying capacities, and individual-level HR patterns, are important drivers of mongoose rabies dynamics^17^. This study provides uniquely detailed information on mongoose HR and fine-scale resource selection in their non-native range, which will contribute to increase our understanding of mongoose rabies epidemiology and can be used to inform the design of rabies control interventions in the Caribbean region. Moreover, it reports unprecedented, quantitative evidence of substantial HR overlap between mongooses and FRDDs. Follow-up studies exploring the nature and frequency of the intra- and interspecific interactions within species HRs would allow better characterizing the implications of the shared use of space between mongooses, the wildlife rabies reservoir, and FRDDs, which are an important vector of mongoose rabies at the One Health interface on Puerto Rico and other Caribbean islands.

## Methods

### Study site

**Figure 5:**
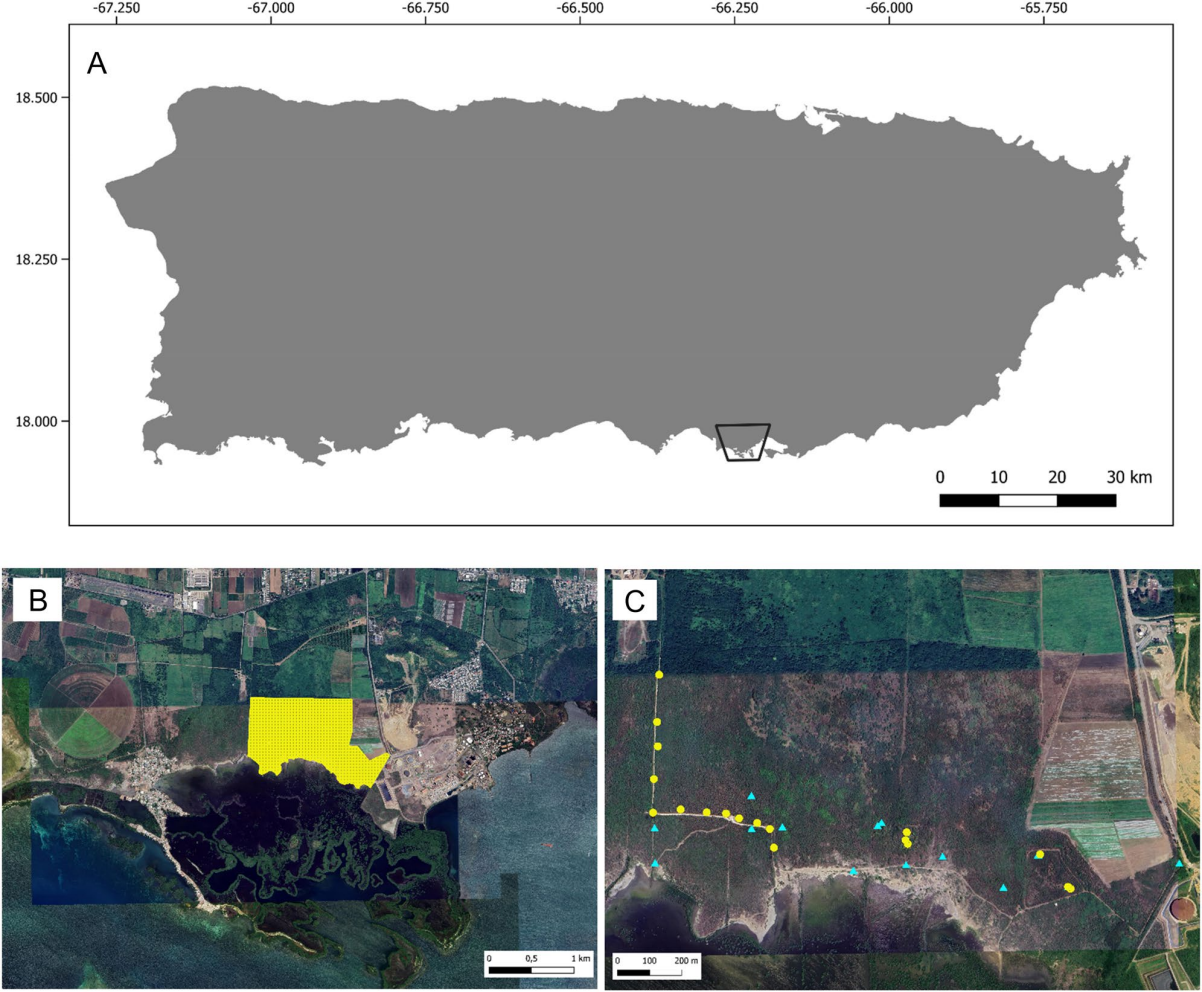
Location of the study site in southern Puerto Rico. Panel (**B**) is an enlargement of the area represented by a black contoured polygon on panel (**A**) and shows the extent of the study site (white shaded polygon). Panel (**C**) displays free-roaming domestic dog (FRDD, *Canis lupus familiaris*; red circles) and small Indian mongoose (*Urva auropunctata*, blue circles) cage traps positioned over the study area to collar animals. Map generated using QGIS v 3.16.4, map data from Google, Image @TerraMetrics 2023.

We conducted this study in the northwest area of the Jobos Bay National Estuarine Research Reserve, Salinas Municipality, Puerto Rico (Fig. 5). The study site area consisted of 443 ha of primarily dry grasslands and pastures (28%) and hay and row crops (27%) based on PRGAP Landcover data^49^. Annual temperatures usually range from 19.5 to 32 °C and monthly rainfall follows a strong seasonal trend, with maximum and minimum rainfall occurring in October (178 mm) and March (25 mm), respectively^50^. In previous capture-mark-recapture (CMR) work conducted between September 1–9 2021 over the study area, we tagged a total of 45 mongooses and estimated mongoose density to 0.97 (95% CI 0.85–1.10) mongooses per hectare (USDA, Unpublished data). In addition, a preliminary camera trapping survey conducted over the study site (see Sauvé et al. In Revision for details) identified 31 unique FRDDs which used the site over a 9-month period.

### Capture and handling

During April 15–20 2022, we live-captured mongooses in cage traps (Tomahawk Trap Company, Hazelhurst, Wisconsin, USA) baited with commercial canned tuna in water^22^. We selected locations for trap placement along dirt roads and walking trails across the site (Fig. 5c). We baited traps daily in the morning, checked them every 3–4 h, and closed them at sunset. We immobilized captured individuals with an intramuscular injection of tiletamine and zolazepam 1:1 (Telazol^®^, Zoetis, Florham, NJ, USA) at a dose of 5 mg/kg^51^. We determined sex and relative age (adult or juvenile) based on size, weight and testicular development in males. While under anesthesia, we fit adult mongooses with a ~ 20 g GPS collar (Litetrack RF-20; Lotek Wireless Inc., Newmarket, Ontario, Canada) and inserted a sterile passive integrated transponder (PIT) tag (AVID Identification Systems Inc., Norco, California, USA) via subcutaneous (SQ) injection between the shoulder blades.

During April 13 and 20 2022, we lived-captured FRDDs using cage traps (Fig. 5c) baited with commercial canned dog food or by attracting docile animals with dog food for manual restraint. We placed cage traps based on the camera study described above, where 10 trail cameras baited with dog food were monitored throughout the study area during the 3 months prior to dog capture. We installed and pre-baited dog traps at the camera sites where FRDDs were most frequently detected (n = 6) for up to 3 weeks prior to capture efforts. We set traps daily in the morning and checked and rebaited them every 2 h until sunset. We closed traps between sunset and sunrise. Because the home ranges of FRDDs from same social groups are likely to be highly similar and correlated, we aimed to track dogs from different social groups. Accordingly, we collared FRDDs that were never observed together based on the camera trap monitoring of the site and personnel observations during capture efforts. Upon capture, sedated FRDDs to be collared with an intramuscular injection of dexmedetomidine (5–10 µg/kg; Dexdomitor^®^, Zoetis) and butorphanol (0.2–0.4 mg/kg; Dolorex^®^; Merck Animal Health, Madison, NJ, USA) for processing. Under sedation, we fit FRDDs with a ~ 330 g GPS collar (Litetrack 330; Lotek Wireless Inc., Newmarket, Ontario, Canada) and inserted a sterile PIT tag as described above for mongooses. We recorded each collar deployment location using a handheld GPS unit (Garmin GPSMAP 64; Olathe, KS, USA).

### Data collection

Since mongooses are strictly diurnal^1,43^, we programmed mongoose collars to record a Swift GPS fix every 30 min from 05:00 to 19:00, which approximately corresponds to one hour after sunrise until one hour before sunset. SWIFT fix devices store a snapshot of GPS ephemeris data and require post-processing to derive location fixes. We programmed dog collars to record a Swift GPS fix every 30 min but extended the recording period to 05:00 to 22:00 to take advantage of the longer battery life afforded by the larger GPS units on the dog collars. We programmed both collar types to emit a VHF beacon signal and to turn on radiofrequency (RF) communication for a 3 h period daily only during late morning. We set the collar mortality function to turn on after 12 h of inactivity. We visited the study area weekly and used a hand-held VHF receiver (R-1000, Communication Specialists Inc., Orange, CA, USA) to attempt re-location of collared animals in proximity and a PinPoint Commander (Lotek Wireless Inc., Newmarket, Ontario, Canada) to remotely download data from the collars.

### Tracking data processing and screening

We imported the raw data from collars on a personal computer via the PinPoint Commander unit and decoded the data using PinPoint Host (v2.15.4.0; Lotek Wireless Inc., Newmarket, Ontario, Canada), by specifying the original deployment location for each collar. We then cleaned GPS tracking data following the steps suggested in^52^. First, we applied temporal filters on tracking data. We removed the initial three hours post-collar deployment to ensure animal recovery from anesthesia. If a collared animal was recaptured in a trap, data from the time it entered the trap until three hours post-release were discarded to avoid potential effects of the capture stress on movement behaviour. When a collar displayed a mortality signal, we excluded the 12 h prior to the time the mortality signal activated. Second, we applied a broad spatial filter by excluding fixes located outside of the Salinas and Guayama municipalities. Exploratory data analysis revealed that this filter would eliminate obvious outliers located in open water or animal locations which were several dozen kilometers away from the study site.

In a third step, we filtered tracks based on data quality using the horizontal dilution of precision (HDOP) attribute, which is related to location error^53^. To determine the appropriate HDOP threshold, we placed two stationary test Litetrack RF-20 collars which were programmed identically to the mongoose collars at known locations in our study site. We left one collar recording 30 min fixes for seven days and the other collar was left for 18 days. For mongoose collars retrieved after a mortality signal was enabled, we measured the location of collar retrieval and added the tracking data following the onset of the mortality event to the stationary test collar data to form a set of known location tracking data. We calculated the Euclidean distance between each recorded location and its associated true location and examined the effect of HDOP on fix location error. We retained GPS fixes within the 95th percentile of HDOP (i.e., < 18), which corresponded to a mean GPS error of 38.0 ± 5.1 m.

Finally, we filtered out biologically implausible movement. Since point outliers or ‘spikes’ are characterized by artificially high incoming and outcoming speeds^54^, we removed GPS fixes having incoming and outcoming speeds higher than the obvious identified gap histograms of speeds from all combined tracks of a given species (which also roughly corresponded to the 99th speed percentile; Supplementary Fig. 3). For mongooses and dogs, we set the speed threshold to 0.25 m s^−1^ and 0.35 m s^−1^, respectively. We decided not to smooth the data because we were interested in individual small-scale movement, and thinning was unnecessary given the relatively low sample size of clean tracks.

### Home range estimation

We estimated mongoose and FRDD individual HR using three estimators: local convex hulls (LoCoH), time dependent local convex hulls (t-LoCoH), and Brownian bridges (Bb). The LoCoH method is a generalization of the minimum convex polygon (MCP) estimator where convex hulls are created for successive animal locations using nearest neighbors^28^. Local hulls can be generated using different approaches, including a fixed number of neighbors (k-LoCoH), a fixed sphere radius (r-LoCoH), or an adaptive sphere of influence (a-LoCoH). We used a-LoCoH since it is considered a better estimator than the two other alternatives because it adequately captures irregular space use by reducing the number of neighbors used in areas where GPS fixes are thin and scattered^28^. The a-LoCoH therefore represents a non-parametric kernel method which implicitly captures time-dependence in movement data. In contrast, t-LoCoH explicitly accounts for time between successive GPS fixes^55^. Bb estimators are based on a random walk process conditioned on time and distance between GPS fixes to estimate the probability that an animal was at a given location at a given time^29^, explicitly incorporating movement data into HR estimation.

We fit a-LoCoH to clean track data using the *adehabitatHR* package^56^ in R (v4.2.1^57^). For each individual track, we calculated the maximal distance (*D*_*max*_) between pairs of points and investigated the relationship between HR area and *a* for *a* values ranging from *D*_*max*_ to 2·*D*_*max*_ using the *LoCoH.a.area* function^58^. We then selected an initial *a* value for each track by graphically identifying the value after which HR area tended to asymptote. We generated a-LoCoH HRs using this initial value and increased a by 10-m increments until there were no more spurious holes in the HR coverage^59^.

We calculated t-LoCoH using the *tlocoh* R package^60^. In t-LoCoH, the scaling parameter *s* controls the degree to which local hulls are local in time and in space (*s* = 0 corresponds to a space only LoCoH estimator, while with *s* = 1 nearest neighbors are defined solely by timing between fixes without regard to space). We identified initial *s* values for each track using the functions *lxy.ptsh.add* and *lxy.plot.sfinder* functions using an interval of interest of 24 h since there were gaps in tracking data at nighttime when collars were not programmed to record fixes. We then identified initial *a* values using the *lhs.plot.isoear* function to minimize the edge to area ratio for 30% to 50% isopleths, as well as the *lhs.plot.isoarea* function to identify *a* values which preceded sharp jumps in HR areas.

We estimated Bb HRs using the *kernelbb* function from the *adehabitatHR* package. We specified the sig2 parameter using the average error from known location collars reported above (38 m), then estimated the sig1 parameter using maximum likelihood (*liker* function in *adehabitatHR*^29^). We extracted 95% and 50% isopleths, which we further interpret as HR and core areas, respectively.

Total HR area for each estimator was calculated in QGIS (v3.16.4^61^) using the NAD 83 UTM Zone 19N projection (ESPG 6662). We quantified intra- and inter-specific HR overlap by calculating the general overlap index (GOI) developed by^42^. For perfectly non-overlapping HRs, GOI = 0, whereas for perfectly overlapping HRs, GOI = 100. In addition, we calculated the pairwise HR overlap proportion among tagged individuals. We compared median pairwise overlap among species and estimators by Mood’s non-parametric median tests using the *Median.test* function from the *agricolea* package^62^.

### Resource selection function

For each of the mongoose tracks, we generated 100 single null models consisting of correlated random walk trajectories based on randomisation of observed turning angles and distances between successive relocations (NMS.randomCRW function from adehabitatLT package^56^). We used these random trajectories as ‘available’ location data to be contrasted to ‘use’ locations consisting of collar GPS fixes in path selection functions (PSF). We extracted fine-scale resolution (15 m) land-cover data (PRGAP Landcover^49^) for paired use and availability locations, and combined rare land-cover categories (Supplementary Table 2). We fitted conditional logit models using the *survival* package^63,64^ in which we defined pair (i.e., point ID within track) as a strata and mongoose ID as a cluster to account for spatial and temporal correlation^58,65^. We set the reference level to the class most represented over the entire study site (dry grasslands and pastures; proportion of area covered: 26.2%).

### Statement of ethical approval

All animal capture and handling were approved by the National Wildlife Research Center’s Institutional Animal Care and Use Committee under approved study protocol QA-3213 and by the University of Montreal Ethics Committee under research document /21-Rech-2076. Capture and handling were also approved by the Puerto Rico Department of Natural and Environmental Resources under scientific collection permit 2021-IC-035. All fieldwork was conducted in accordance with approved protocols and existing regulation, and this study is reported in accordance with ARRIVE guidelines.

### Supplementary Information

Supplementary Information.

## Data Availability

The datasets generated and/or analysed in the current study are available from the corresponding author on reasonable request.
