# Supplementary material for: Home range overlap between small Indian mongooses and free roaming domestic dogs in Puerto Rico: implications for rabies management
Source: Sci Rep. 2023 Dec 22;13:22944. doi: 10.1038/s41598-023-50261-7 (PMC10746706; doi:10.1038/s41598-023-50261-7)
Supplement: Supplementary file 1 — Supplementary Information. [file 41598_2023_50261_MOESM1_ESM.docx]

# Supplementary information

**Supplementary figure 1** – Correlation between the three different home range (HR) estimators (local convex hulls (a-LoCoH), time-dependant local convex hulls (a-t-LoCoH), and Brownian bridge 95% isopleth (Bb)) for 19 small Indian mongooses (*Urva auropunctata*, top row) and five free roaming domestic dogs (FRDD, *Canis lupus familiaris*; bottom row) equipped with GPS collars. The grey dashed line illustrates the linear relationship between estimators, where all slopes significantly differ from zero.


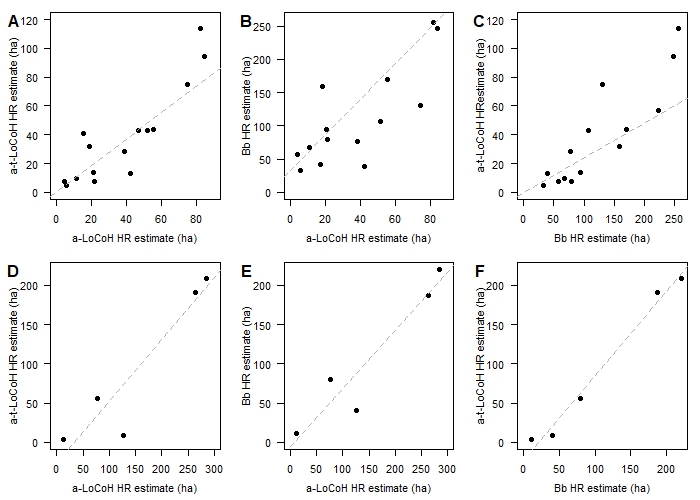


**Supplementary table 1** – Pairwise intra- and interspecific percent home range (HR) overlap among tagged mongooses (n=19) and free-roaming domestic dogs (FRDD; n=5) . HR derived from two estimators were used to compute pairwise overlap: the time-dependant local convex hull (a-t-LoCoH) 95% isopleth, and the 95% Brownian bridge.

| Individual A | Individual B | a-t-LoCoH iso 95 | Brownian bridge 95% |  |
| --- | --- | --- | --- | --- |
| Mongoose-Mongoose (intraspecific) | | | | |
| M01 | M02 | 0 | 1.23 |  |
| M01 | M03 | 0 | 0 |  |
| M01 | M04 | 0 | 57.35 |  |
| M01 | M05 | 86.97 | 85.38 |  |
| M01 | M06 | 2.69 | 17.72 |  |
| M01 | M07 | 0 | 9.99 |  |
| M01 | M08 | 55.17 | 56.83 |  |
| M01 | M09 | 0.57 | 6.87 |  |
| M01 | M10 | 0 | 27.32 |  |
| M01 | M11 | NA | 24.41 |  |
| M01 | M12 | 0 | 1.79 |  |
| M01 | M13 | 0 | 27.3 |  |
| M01 | M14 | 46.98 | NA |  |
| M01 | M15 | 0 | 0 |  |
| M01 | M16 | 0 | 51.56 |  |
| M01 | M17 | 0 | 2.57 |  |
| M02 | M03 | 57.2 | 75.41 |  |
| M02 | M04 | 0 | 5.82 |  |
| M02 | M05 | 0 | 3.25 |  |
| M02 | M06 | 0 | 0.15 |  |
| M02 | M07 | 57.35 | 89.76 |  |
| M02 | M08 | 0 | 0.8 |  |
| M02 | M09 | 71.96 | 74.87 |  |
| M02 | M10 | 44.91 | 31.79 |  |
| M02 | M11 | NA | 8.74 |  |
| M02 | M12 | 42.56 | 34.93 |  |
| M02 | M13 | 0 | 1.14 |  |
| M02 | M14 | 0 | NA |  |
| M02 | M15 | 8.89 | 14.97 |  |
| M02 | M16 | 9.92 | 31.63 |  |
| M02 | M17 | 15.19 | 24.68 |  |
| M03 | M04 | 0 | 3.51 |  |
| M03 | M05 | 0 | 0 |  |
| M03 | M06 | 0 | 0 |  |
| M03 | M07 | 37.94 | 82.76 |  |
| M03 | M08 | 0 | 0 |  |
| M03 | M09 | 81.07 | 91.58 |  |
| M03 | M10 | 7.49 | 19.79 |  |
| M03 | M11 | NA | 6.65 |  |
| M03 | M12 | 33.51 | 36.24 |  |
| M03 | M13 | 0 | 0 |  |
| M03 | M14 | 0 | NA |  |
| M03 | M15 | 5.08 | 13.5 |  |
| M03 | M16 | 6.49 | 26.64 |  |
| M03 | M17 | 8.96 | 23.19 |  |
| M04 | M05 | 0 | 56.34 |  |
| M04 | M06 | 0 | 1.1 |  |
| M04 | M07 | 0 | 36.91 |  |
| M04 | M08 | 0 | 36.98 |  |
| M04 | M09 | 43.47 | 31.91 |  |
| M04 | M10 | 17.55 | 44.18 |  |
| M04 | M11 | NA | 37.52 |  |
| M04 | M12 | 0 | 20.85 |  |
| M04 | M13 | 0 | 9.34 |  |
| M04 | M14 | 74.08 | NA |  |
| M04 | M15 | 0 | 0 |  |
| M04 | M16 | 57.49 | 92.1 |  |
| M04 | M17 | 0 | 21.9 |  |
| M05 | M06 | 26.91 | 23.26 |  |
| M05 | M07 | 0 | 3.1 |  |
| M05 | M08 | 40.5 | 36.27 |  |
| M05 | M09 | 0.07 | 2.19 |  |
| M05 | M10 | 0 | 8.81 |  |
| M05 | M11 | NA | 11.53 |  |
| M05 | M12 | 0 | 0.4 |  |
| M05 | M13 | 17.37 | 33.59 |  |
| M05 | M14 | 16.9 | NA |  |
| M05 | M15 | 0 | 0 |  |
| M05 | M16 | 0 | 23.44 |  |
| M05 | M17 | 0 | 0.48 |  |
| M06 | M07 | 0 | 2.01 |  |
| M06 | M08 | 1.75 | 53.45 |  |
| M06 | M09 | 0 | 0 |  |
| M06 | M10 | 0 | 0 |  |
| M06 | M11 | NA | 0 |  |
| M06 | M12 | 0 | 0 |  |
| M06 | M13 | 45.51 | 89.85 |  |
| M06 | M14 | 0 | NA |  |
| M06 | M15 | 0 | 0 |  |
| M06 | M16 | 0 | 0 |  |
| M06 | M17 | 0 | 0 |  |
| M07 | M08 | 0 | 0 |  |
| M07 | M09 | 83.28 | 74.44 |  |
| M07 | M10 | 20.69 | 27.2 |  |
| M07 | M11 | NA | 8.31 |  |
| M07 | M12 | 58.57 | 35.2 |  |
| M07 | M13 | 0 | 0.28 |  |
| M07 | M14 | 0 | NA |  |
| M07 | M15 | 11.68 | 11.54 |  |
| M07 | M16 | 13.01 | 34.06 |  |
| M07 | M17 | 21.14 | 23.18 |  |
| M08 | M09 | 0.11 | 0 |  |
| M08 | M10 | 0 | 7.97 |  |
| M08 | M11 | NA | 20.89 |  |
| M08 | M12 | 0 | 0 |  |
| M08 | M13 | 6.15 | 39.52 |  |
| M08 | M14 | 12.6 | NA |  |
| M08 | M15 | 0 | 0 |  |
| M08 | M16 | 0 | 32.06 |  |
| M08 | M17 | 0 | 0 |  |
| M09 | M10 | 21.56 | 23.57 |  |
| M09 | M11 | NA | 7.8 |  |
| M09 | M12 | 35.05 | 40.6 |  |
| M09 | M13 | 0 | 0 |  |
| M09 | M14 | 2.76 | NA |  |
| M09 | M15 | 4.43 | 13.05 |  |
| M09 | M16 | 24.46 | 35.71 |  |
| M09 | M17 | 8.62 | 26.22 |  |
| M10 | M11 | NA | 21.86 |  |
| M10 | M12 | 12.54 | 21.48 |  |
| M10 | M13 | 0 | 0 |  |
| M10 | M14 | 20.32 | NA |  |
| M10 | M15 | 3.14 | 13.56 |  |
| M10 | M16 | 15.28 | 53.98 |  |
| M10 | M17 | 6.02 | 23.38 |  |
| M11 | M12 | NA | 36.6 |  |
| M11 | M13 | NA | 4.64 |  |
| M11 | M15 | NA | 10.03 |  |
| M11 | M16 | NA | 100 |  |
| M11 | M17 | NA | 41.18 |  |
| M12 | M13 | 0 | 0 |  |
| M12 | M14 | 0 | NA |  |
| M12 | M15 | 5 | 21.85 |  |
| M12 | M16 | 30.43 | 69.83 |  |
| M12 | M17 | 22.82 | 59.23 |  |
| M13 | M14 | 0 | NA |  |
| M13 | M15 | 0 | 0 |  |
| M13 | M16 | 0 | 9.04 |  |
| M13 | M17 | 0 | 0 |  |
| M14 | M15 | 0 | NA |  |
| M14 | M16 | 12.29 | NA |  |
| M14 | M17 | 0 | NA |  |
| M15 | M16 | 0 | 42.2 |  |
| M15 | M17 | 7.1 | 51.46 |  |
| M16 | M17 | 15.82 | 39.61 |  |
| Dog-dog (intrapsecific) | | | |  |
| D01 | D02 | 0 | 0.17 |  |
| D01 | D03 | 0 | 0 |  |
| D01 | D04 | 0 | 0 |  |
| D01 | D05 | 0 | 0 |  |
| D02 | D03 | 33.28 | 27.35 |  |
| D02 | D04 | 0 | 1.53 |  |
| D02 | D05 | 0 | 2.36 |  |
| D03 | D04 | 0 | 0 |  |
| D03 | D05 | 0 | 8.64 |  |
| D04 | D05 | 0.13 | 10.92 |  |
| Mongoose-dog (interspecific) | | | | |
| D01 | M01 | 0 | 0 | |
| D01 | M02 | 18.84 | 28.71 | |
| D01 | M03 | 54.15 | 32.41 | |
| D01 | M04 | 0 | 0 | |
| D01 | M05 | 0 | 0 | |
| D01 | M06 | 0 | 0 | |
| D01 | M07 | 31.29 | 24.7 | |
| D01 | M08 | 0 | 0 | |
| D01 | M09 | 39.73 | 31.34 | |
| D01 | M10 | 0 | 0 | |
| D01 | M11 | NA | 0 | |
| D01 | M12 | 26.21 | 30.47 | |
| D01 | M13 | 0 | 0 | |
| D01 | M14 | 0 | NA | |
| D01 | M15 | 0 | 24.87 | |
| D01 | M16 | 0.54 | 5.9 | |
| D01 | M17 | 2.3 | 15.85 | |
| D02 | M01 | 0 | 0 | |
| D02 | M02 | 7.17 | 18.08 | |
| D02 | M03 | 0.23 | 13.83 | |
| D02 | M04 | 0 | 0 | |
| D02 | M05 | 0 | 0 | |
| D02 | M06 | 0 | 0 | |
| D02 | M07 | 7.58 | 13.94 | |
| D02 | M08 | 0 | 0 | |
| D02 | M09 | 1.07 | 12.05 | |
| D02 | M10 | 0.32 | 14.31 | |
| D02 | M11 | NA | 0 | |
| D02 | M12 | 0 | 4.5 | |
| D02 | M13 | 0 | 0 | |
| D02 | M14 | 0 | NA | |
| D02 | M15 | 0.19 | 41.67 | |
| D02 | M16 | 0 | 1.11 | |
| D02 | M17 | 0 | 4.05 | |
| D03 | M01 | 0 | 0 | |
| D03 | M02 | 1.49 | 4.94 | |
| D03 | M03 | 0 | 4.46 | |
| D03 | M04 | 0 | 0 | |
| D03 | M05 | 0 | 0 | |
| D03 | M06 | 0 | 0 | |
| D03 | M07 | 1.47 | 3.81 | |
| D03 | M08 | 0 | 0 | |
| D03 | M09 | 0 | 4.24 | |
| D03 | M10 | 0 | 2.92 | |
| D03 | M11 | NA | 0 | |
| D03 | M12 | 0 | 0 | |
| D03 | M13 | 0 | 0 | |
| D03 | M14 | 0 | NA | |
| D03 | M15 | 0 | 9.57 | |
| D03 | M16 | 0 | 0 | |
| D03 | M17 | 0 | 0 | |
| D04 | M01 | 0.62 | 6.69 | |
| D04 | M02 | 4.8 | 3.79 | |
| D04 | M03 | 0 | 1.45 | |
| D04 | M04 | 0 | 0 | |
| D04 | M05 | 0 | 9.37 | |
| D04 | M06 | 0 | 13.2 | |
| D04 | M07 | 0 | 2.25 | |
| D04 | M08 | 0 | 3.34 | |
| D04 | M09 | 0 | 1.53 | |
| D04 | M10 | 41.05 | 10.62 | |
| D04 | M11 | NA | 2.56 | |
| D04 | M12 | 0 | 1.21 | |
| D04 | M13 | 0 | 5.47 | |
| D04 | M14 | 16.33 | NA | |
| D04 | M15 | 0 | 0 | |
| D04 | M16 | 0 | 0.97 | |
| D04 | M17 | 0 | 2.24 | |
| D05 | M01 | 58.6 | 45.76 | |
| D05 | M02 | 0 | 3.75 | |
| D05 | M03 | 0 | 0.39 | |
| D05 | M04 | 8.46 | 18.62 | |
| D05 | M05 | 87.58 | 68.34 | |
| D05 | M06 | 100 | 100 | |
| D05 | M07 | 0 | 0.6 | |
| D05 | M08 | 82.68 | 55.27 | |
| D05 | M09 | 0 | 0.37 | |
| D05 | M10 | 0 | 0.32 | |
| D05 | M11 | NA | 11.49 | |
| D05 | M12 | 0 | 0 | |
| D05 | M13 | 100 | 96.32 | |
| D05 | M14 | 9.3 | NA | |
| D05 | M15 | 0 | 0.85 | |
| D05 | M16 | 0 | 6.77 | |
| D05 | M17 | 0 | 0 | |

**Supplementary figure 2** – Brownian bridge home range estimate (yellow polygon) and full tracking record (blue track) from male free-roaming domestic dog (FRDD) #D05. The home range was estimated based on the tracking data clipped to correspond to the mongoose collar deployment period during this study (April 16^th^ – June 23^rd^, 2022). However, the complete track for D05 extended to November 18^th^, 2022. During the period following mongoose tracking, D05 dispersed to a new area location ~10 km West of his initial home range centroid.


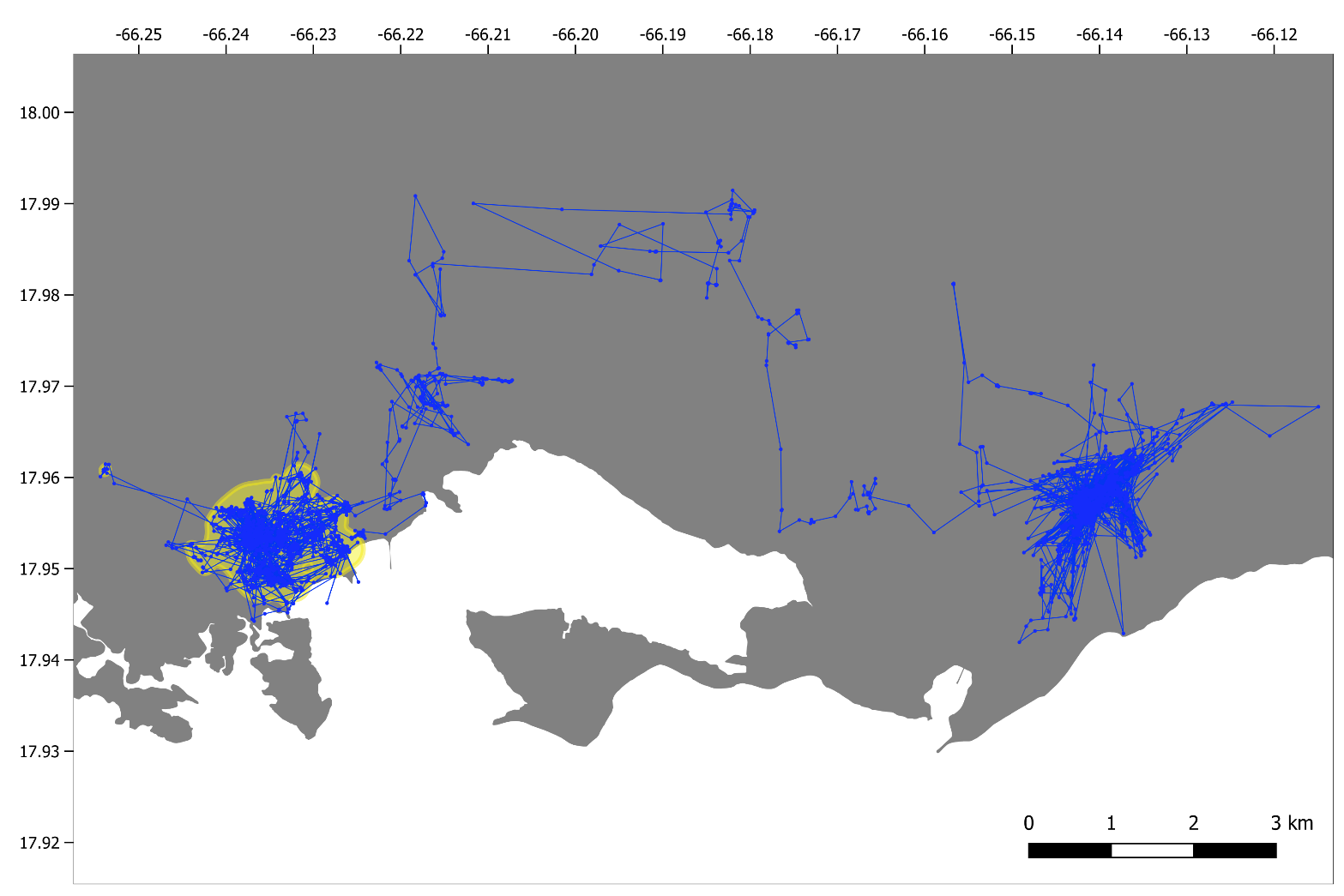


**Supplementary figure 3**– Distribution of mongoose (A) and free-roaming domestic dog (FRDD; B) speed calculated between successive locations recorded by GPS collars. Abscissa axis of histograms were truncated to improve visual clarity, but there were 26 unshown mongoose locations (maximum speed: 4.3 m·s^-1^) and 310 unshown FRDD locations (maximum speed: 11.6 m·s^-1^). The 99^th^ percentile (grey dashed line), which corresponded to 0.25 and 0.35 m·s^-1^ for mongooses and FRDDs, respectively, was used to filter out biologically implausible movement from tracking data.


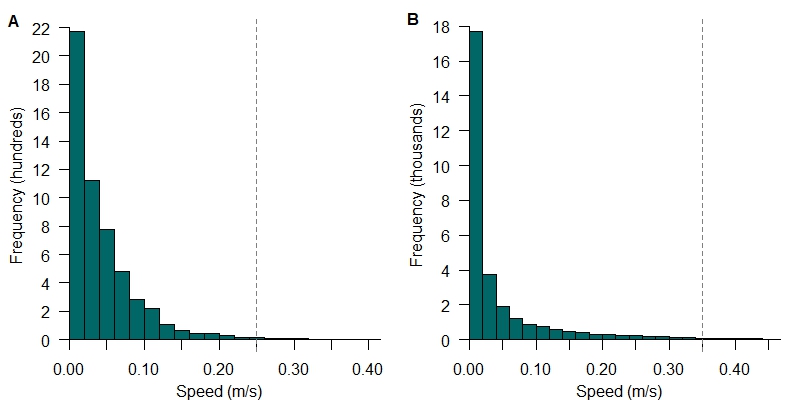


Supplementary table 2 – Definition of combined land-cover classes based on thematic resolution simplification for rare PRGAP land-cover classes (Gould et al. 2008) over the study site. Numbers refer to class ID defined in PRGAP metadata (file:///C:/Users/p0918148/Documents/PhD/shapefiles/PR_Landcover_GAP_2000_15m_res/metadata_prgap_landcover_original.html; accessed May 2023).

| **Simplified land-cover category** | **Categories used in resampling** |
| --- | --- |
| Secondary lowland dry noncalcareous semideciduous forest | 13. Mature secondary lowland dry noncalcareous semideciduous forest  14. Young secondary lowland dry noncalcareous semideciduous forest |
| Grasslands and pastures | 48. Dry grasslands and pastures  50. Moist grasslands and pastures |
| Agricultural vegetation | 55. Hay and row crops  56. Woody agriculture and plantations, palm plantations |
| Sandy/mixed sand and gravel beaches, san and mudflats | 59. Fine to coarse sandy beaches, mixed sand and gravel beaches  61. Salt and mud flats |
| Artificial, riparian or other natural barrens | 60. Riparian and other natural barrens  65. Artificial barrens |
